# Using enzyme activities as an indicator of soil fertility in grassland - an academic dilemma

**DOI:** 10.3389/fpls.2023.1175946

**Published:** 2023-07-07

**Authors:** Li Wang, Chantal Hamel, Peina Lu, Junying Wang, Dandi Sun, Yijia Wang, Soon-Jae Lee, Gary Y. Gan

**Affiliations:** ^1^ College of Life and Environmental Sciences, State & Local Joint Engineering Research Center for Ecological Treatment Technology of Urban Water Pollution, Zhejiang Provincial Key Lab for Water Environment and Marine Biological Resources Protection, Zhejiang Provincial Collaborative Innovation Center for Tideland Reclamation and Ecological Protection, Wenzhou University, Wenzhou, Zhejiang, China; ^2^ Soil Microbiology Scientist, Commerciale, Rivière-à-Pierre, QC, Canada; ^3^ State Key Laboratory of Aridland Crop Science, Gansu Agricultural University, Lanzhou, China; ^4^ Department of Ecology and Evolution, University of Lausanne, Lausanne, Switzerland; ^5^ Agroecosystems, the uBC-Soil Group, Kelowna, BC, Canada

**Keywords:** ecosystem functions and services, ecosystem sustainability and resilience, microbial biomass, soil biochemical property, physiochemical property

## Abstract

Grasslands play an important role in conserving natural biodiversity and providing ecosystem functions and services for societies. Soil fertility is an important property in grassland, and the monitoring of soil fertility can provide crucial information to optimize ecosystem productivity and sustainability. Testing various soil physiochemical properties related to fertility usually relies on traditional measures, such as destructive sampling, pre-test treatments, labor-intensive procedures, and costly laboratory measurements, which are often difficult to perform. However, soil enzyme activity reflecting the intensity of soil biochemical reactions is a reliable indicator of soil properties and thus enzyme assays could be an efficient alternative to evaluate soil fertility. Here, we review the latest research on the features and functions of enzymes catalyzing the biochemical processes that convert organic materials to available plant nutrients, increase soil carbon and nutrient cycling, and enhance microbial activities to improve soil fertility. We focus on the complex relationships among soil enzyme activities and functions, microbial biomass, physiochemical properties, and soil/crop management practices. We highlight the biochemistry of enzymes and the rationale for using enzyme activities to indicate soil fertility. Finally, we discuss the limits and disadvantages of the potential new molecular tool and provide suggestions to improve the reliability and feasibility of the proposed alternative.

## 1 Introduction

Grasslands are the most widely distributed terrestrial ecosystems in the world, playing a critical role in conserving natural biodiversity (Klein et al., 2020; Wahdan et al., 2021), providing essential ecosystem services for billions of people’s livelihoods (Bengtsson et al., 2019; Li M. et al., 2022), and helping mitigate the anthropogenically-driven impacts of climate change (Han et al., 2018; Dong et al., 2020). Research in recent decades has concentrated on the development of strategies to rejuvenate degraded grasslands to enhance sustainability, with excellent scientific findings documented on the impacts of environmental factors (such as climate change) (Han et al., 2018; Nandintsetseg et al., 2021) and anthropogenic activities (such as grazing and fertilization) (Chen et al., 2019; Luo et al., 2019; Wu et al., 2021) on grassland ecosystem functions and services. Enzymes – a critical biochemical component in soil, play an important role in catalyzing biochemical processes to convert soil organic matter (SOM) to available nutrients, increase carbon (C) and nutrient cycling, and enhance microbial activities to improve the soil environment (Chuan et al., 2020; Ullah et al., 2021; Dong et al., 2022; Yang Y. et al., 2022). These enzyme-induced biochemical activities are closely correlated to other physiochemical properties in grassland soils. Enzyme activity reflects the intensity of biochemical reactions, which plays a critical role in maintaining the health, stability, and resilience of ecosystems.

‘Soil fertility’ is defined as the ability of soil to provide the conditions required for plant growth where the physical, chemical, and biological processes act together to provide nutrients, water, aeration, and stability to the plant, as well as freedom from any substances that may inhibit growth (Stockdale et al., 2002). This definition covers the key conceptual attributes of fertility and has been commonly used in agricultural science. Food and Agriculture Organization of the UN defines soil fertility as the ability of soil to sustain plant growth by providing essential plant nutrients and favorable chemical, physical, and biological characteristics as a habitat for plant growth (Montanarella, 2015). Some other scholars define soil fertility as: (1) the soil ability to supply plant nutrients in the right quantities and qualities over a sustained period, (2) inherent capacity of soil to supply nutrients to plants in adequate amounts and in suitable proportions, (3) as a soil’s potential to create favorable chemical, physical, and biological conditions and provide all the essential nutrients to support plant growth Alfen (2014), (4) much narrowly the ability of the soil to provide nutrients that are essential for plant growth (Anonymous, 2023), (5) ability to provide the foundation for nutritious food production and resilient and sustainable livelihoods (Stewart et al., 2019), (6) soil fertility can be defined as the capacity of the soil to provide plants with sufficient available nutrients to produce crops (Abbott and Murphy, 2003), (7) capacity of soil to provide physical, chemical and biological needs for the growth of plants for productivity, reproduction and quality (Bayu, 2020), or (8) the quality of a soil that enables it to provide nutrients in adequate amounts and in proper balance for the growth of specific plants or crops (Buresh et al., 1997).

Soil fertility is a key factor affecting grassland ecosystem functions and services. However, the measurement of soil fertility is complex. The currently available methods are more or less standardized (Anonymous, 2020), but some procedures are labor-intensive (e.g., non-invasive soil water content), and many analytic procedures are costly (e.g., SOC, soil organic carbon). An interesting question is raised in the research community: can we use alternative methods to estimate and assess soil fertility more efficiently than the current methods? One of the alternatives that have been in the debate is whether or not soil enzyme activity can be used as a biological indicator of soil fertility. The notion is based on the understanding that (1) soil enzyme activities are closely correlated with soil fertility-related physiochemical properties, and they are more sensitive to natural and anthropogenic disturbances and have the potential to provide an integrative biological assessment of soil fertility (Alkorta et al., 2003); and (2) the measurement of soil enzyme activity is relatively easier (Piotrowska-Długosz et al., 2021), faster (Piotrowska-Długosz et al., 2021), and lower in cost (Bueis et al., 2018) than that of various soil fertility parameters. However, using soil enzyme activities as an indicator of soil fertility has many limitations. For example, soil enzymatic activities in degraded soil express in a wide range compared to the undegraded control, with a range of 37-260% for phosphomonoesterase activity, 16-250% for β-glucosidase, and 24-250% for urease and dehydrogenase (Trasar-Cepeda et al., 2000). The wide expression of enzymatic activities makes interpretations difficult. For some enzymes, the associations between enzyme activity and soil physicochemical properties are weak, nonexistent, or unknown (Lee et al., 2020), while for the others, their activities in soil differ substantially with changes in soil environmental factors such as temperature and moisture (Wolińska and Stepniewska, 2012).

The above arguments lead to a dilemma, a problem to choose between the direct measurements of the various soil fertility-related parameters as have been and the assessment of enzymatic activities to indicate soil fertility. An in-depth understanding of the features of enzymes and the roles they play in serving the grassland ecosystem is required for assessing the value of using soil enzyme activities as an indicator of soil fertility. In this mini-review, we briefly summarize the basic biochemical characteristics of soil enzymes and their functioning in grassland ecosystems and highlight the relationships among enzyme activities, soil microbial biomass, and physiochemical properties. Finally, we discuss the feasibility and potential of using enzyme activity as an indicator of soil fertility.

## 2 Biochemistry of enzymes

**Figure 1:**
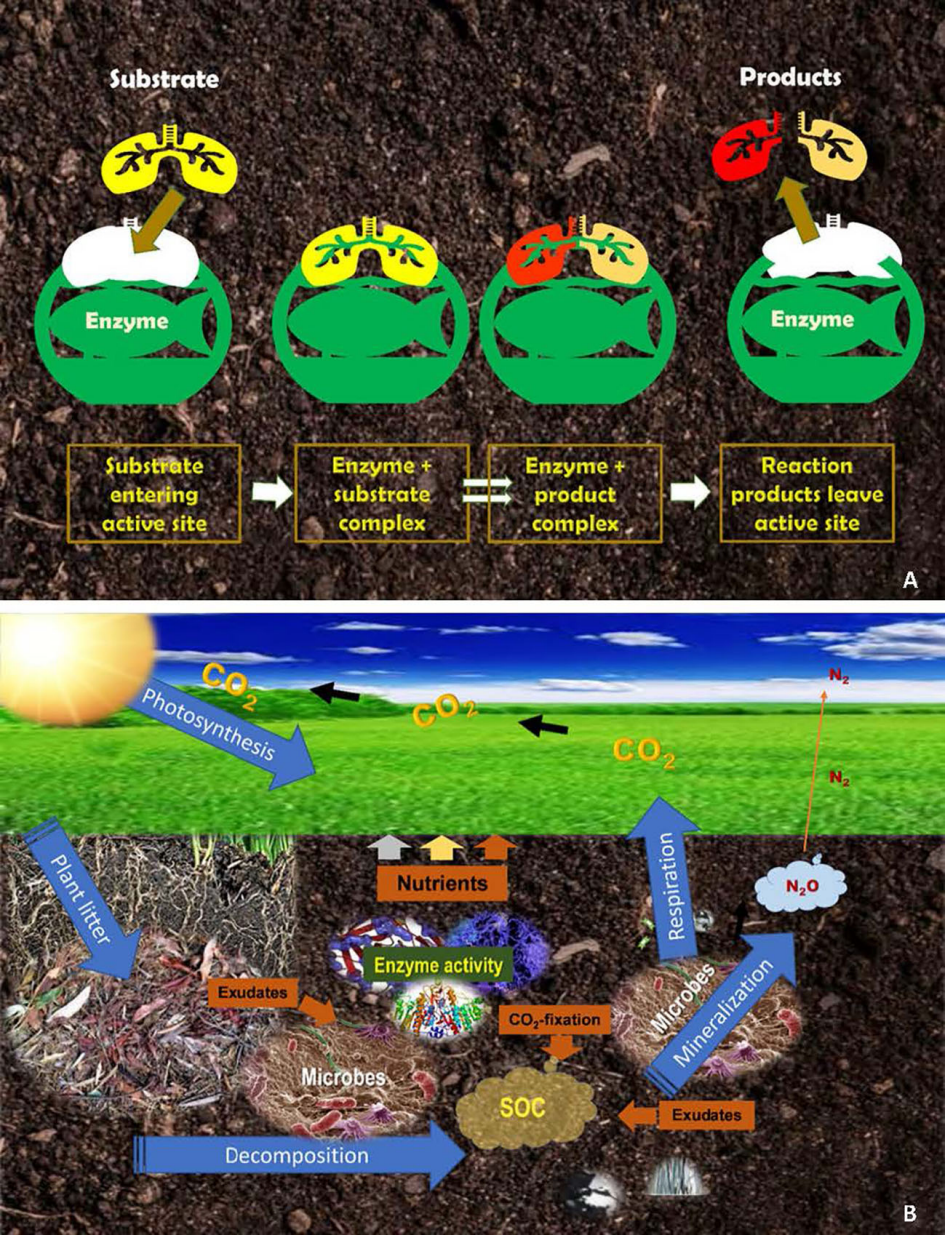
An illustration of the reduced fit between an enzyme’s active site and the substrate **(A)** and the functioning of enzymes and microbial communities in the biochemical processes of carbon and nutrient cycling in the grassland ecosystem **(B)**.

Enzymes play a significant role in the cycling of carbon and the other nutrients in grassland ecosystems, and therefore, it is important to understand how an enzyme acts and reacts biochemically in response to soil/crop management practices. An enzyme is a molecule produced in small amounts by cells in living organisms to carry out a biochemical reaction (Anonymous, 2022). A substance that reacts upon the active site of an enzyme is called a substrate. The active site is a place where the catalytic action happens (**Figure 1A**). In a biochemical reaction, the active site of the enzyme and the substrate form an enzyme-substrate complex. The substrate is then transformed into one or more products that are released from the active site of the complex. For instance, the enzyme carbohydrase breaks down carbohydrates (substrate) and releases sugars (products) that can then be used as an energy source.

Based on the site of the enzyme works, enzymes can be categorized as intracellular and extracellular enzymes. Intracellular enzymes are synthesized by the cells and retained within the cytoplasm, chloroplasts, mitochondria, and nucleus for cellular biochemical reactions, whereas extracellular enzymes are secreted and function outside the cell, such as digestive enzymes (pepsin and salivary amylase). In the grassland ecosystem, there are plenty of types of enzymes (Hillel, 2005) including the amylase, glucosidase, cellulase, chitinase, dehydrogenase, phosphatase, protease, and urease. Most soil enzymes come from root exudates and microorganisms, and decompose soil litter and other SOM. Other important enzymes located inside soil organisms mineralize organic molecules, releasing nutrients in plant-available forms. Enzyme activity is measured as the amounts of substrate transformed or product produced per unit of time. Enzyme activity determines the rate of biological processes occurring within living organisms in the case of endoenzymes, or the soil in the case of exoenzymes. Various tools can be used to measure enzymatic activity, including mass spectrometry, electrochemistry, capillary electrophoresis, radiometric methods, colorimetric analysis, and fluorescence methods (Erel, 2005; Bailey et al., 2011; Niu et al., 2019). Also, some specialized methods for measuring enzyme activities in real time have been in use, such as the catalytic activity of TEM1-β-lactamase inside living cells (Zotter et al., 2017), single-cell assays of enzyme activity (Kovarik and Allbritton, 2011), ultrasensitive detection of enzymatic activity using single molecule arrays (Wang X. et al., 2020), kinase activity in cell lysates (Yi et al., 2018), and enzyme activity at *in vivo* concentrations using silver nanoparticles (Moore et al., 2004). Many methods are still evolving.

## 3 Enzyme activities and functions in the grassland ecosystem

### 3.1 Enzyme functioning

In grassland ecosystems, the central, most important role enzymes play is the oxidization of organic matter. Organic debris entering the soil undergo enzyme-driven oxidation – a reaction by which complex organic compounds are oxidized into smaller compounds and molecules, and ultimately, into usable elements or waste products. In the process, the energy contained in decomposing organic molecules and some enzyme products are captured to sustain the metabolism of soil organisms, but by-products including plant nutrients are also released into soil and recycled (**Figure 1B**). In agricultural soils, the mineralization of SOM is mainly driven by fungi, bacteria, and earthworms. Thus, the enzymatic activity of these organisms is important in regulating the flux of energy and nutrients sustaining the grassland ecosystem.

In the soil environment, one enzyme may function for a specific biochemical reaction. For example, the enzymes β-glucosidase, invertase, and hydrolase are mainly responsible for the cycling of C in soils, whereas urease, alkaline phosphatase, and sulfatase are responsible for the cleavage of N, P, and S from organic compounds and consequently, are responsible for replenishing the soil solution with mineral N, P, and S forms that plants can use. In grassland ecosystems, an increase in enzyme activity usually means an increase in the contact area between the enzyme and the substrates, which accelerates the bioprocess of material transformation. A high rate of enzyme activity may be due to the abundance of the substrates, but not always true; for example, phosphatase activity is increased by the scarcity of available P and inhibited by its abundance. There are many individual enzymes in grassland ecosystems, each having different functions. The detailed functions are summarized in **Table 1**.

**Table 1:** Main enzymes and their functions in grasslands.

Enzyme	Reaction	Compounds obtained from the reaction	Main functions in grassland ecosystems	References
β-glucosidase	Hydrolyzes glycosides and oligosaccharides	Glucose	Sustains soil life through the provision of C and energy sources	(Wang et al., 2019a; Chuan et al., 2020; Du et al., 2020; Li Q. et al., 2021; Saha et al., 2021; Hu et al., 2022; Li N. et al., 2022)
Invertase	Catalyzes the process of decomposing sucrose	Fructose and glucose	Sustains soil life through the provision of C and energy sources	(Veriato Coura et al., 2020; Liao et al., 2023; Liu et al., 2023; Pan et al., 2023)
Catalase	Decomposes hydrogen peroxide	Water and oxygen	Protect living cells from oxidative damage	(Hu et al., 2022; Li J. et al., 2022; Li M. et al., 2022)
Dehydrogenases	Catalyzes numerous oxidation-reduction reactions	Various, often aldehydes and protons	Allows various biochemical reactions	(Marcos et al., 2020; Kaur, 2021; Saha et al., 2021; Hu et al., 2022; Li N. et al., 2022)
Amylase	Hydrolyses starch	Sugars	Sustains soil life through the provision of C and energy sources	(Veriato Coura et al., 2020; Wang H. Y. et al., 2020; Krishnan et al., 2022; Ozcan et al., 2022)
Urease	Hydrolyses urea	Ammonia and carbon dioxide	Provides plant available N	(Wang C. et al., 2020; Wang et al., 2021; Ekwunife et al., 2022)
Phosphatase	Hydrolyses phosphor-ester bonds	Phosphate ion	Provides plant available P	(Yang et al., 2016; Liao et al., 2020; Wahdan et al., 2021; Xie et al., 2021)
Sulfatase	Hydrolyses sulfate-ester bonds	Sulfate ion	Provides plant available S	(Tscherko et al., 2004; dos Santos et al., 2022)
Cellulases	Hydrolyses 1,4-beta-glucosidic linkages	Mono- and oligo-saccharides	Sustains soil life through the provision of C and energy sources	(Iqbal et al., 2021; Liu et al., 2022; Wang et al., 2022)
Chitinase	Hydrolyzes the chitin polymers of arthropods’ exoskeleton and fungal cell wall	Low molecular weights multimers	Triggers plant defense and first step in the production of mineral N and C from chitin	(Bazghaleh et al., 2016; Khan et al., 2019; Sousa et al., 2019; Kim et al., 2022; Muñoz et al., 2022)

### 3.2 Relationship between enzyme activity and microbial biomass

In grassland ecosystems, soil microbial activity drives the C and N cycles in which enzymes act as the catalyst while microbial biomass serves as the precursor. Thus, soil enzymes and microbial biomass are two important microbiological properties that play a crucial role in the process of converting the active components of SOM to nutrient elements contributing to soil fertility.

The activity of soil enzymes and microbial biomass is associated with soil physiochemical properties in a complex manner. The ratio of the extracellular enzyme (Yang et al., 2020) and their activities (Dong et al., 2021) are related to the demand for C, N, and P resources by microorganisms. For example, the elemental composition of soil microbial biomass is closely associated with specific enzyme stoichiometry in C digestion (Yuan et al., 2019). However, the relationship between biomass composition and enzyme activity varies with time and space. In a moderately degraded grassland, soil microbial biomass C content was positively correlated with invertase activity, while soil microbial biomass N content was negatively correlated with soil invertase activity. In desert areas, soil microbial biomass C and N did not correlate with soil urease or invertase, but in a degraded alpine grassland, soil microbial biomass C and N were correlated with soil invertase and urease activities. In a semi-natural grassland, elevating ozone changed the enzyme activity, but not the function of the soil microbial community (Wang J. et al., 2019).

The above research observations show that there is a complex relationship between soil microorganisms and enzyme activity. An increase in soil microbial biomass can increase soil enzyme activities, but not always, because the relationship can be influenced by many other factors. For example, microbial community composition, and enzyme activity are directly and indirectly related to plant biotype (Cao et al., 2019), plant species composition (Chuan et al., 2020), the primary and net productivities of grasslands (Liu et al., 2020), microbial community structure (Li Q. et al., 2021), and SOC concentration (Li Y. et al., 2019). Often enzyme activities and microbial biomass composition are more sensitive to environmental conditions (e.g., soil temperature, moisture, pH) and human/animal activities (e.g., grazing and trampling, and land use change) (Armbruster et al., 2021; dos Santos et al., 2022), than to the physicochemical properties such as nutrient concentration.

### 3.3 Enzyme activity and grassland management practices

Grassland management practices such as grazing, N fertilization, mowing, and the conversion to cropland influence soil microbial biomass and enzyme activities as follows.

1) Grazing

Grazing influences the productivity of grassland ecosystems by modifying aboveground and belowground plant biomass, soil microbial biomass, and nutrient dynamics. Yang X. et al. (2022) conduct a meta-analysis using 934 paired observations from 69 studies to assess the effect of grazing intensity (low, medium, heavy grazing) on soil microbial biomass and community composition in steppe ecosystems. They find that heavy grazing reduces the numbers of bacteria, fungi, and actinomycetes by 30-92% as compared to grazing exclusion, which translates in to a 14-36% reduction in microbial biomass. In contrast, grazing exclusion for a short period (4 years) rapidly increases soil microbial biomass through the production of more substrates for microbial activities, and increases the activities of soil enzymes (Du et al., 2020). In well-managed grazing systems, the activities of β-glucosidase in the soil are more sensitive to changes in practices than microbial biomass carbon and respiration (dos Santos et al., 2022). However, the impacts of grazing on soil microbial biomass and enzyme activity are inconsistent from one study to another. In a semiarid grassland with different grazing intensities, Rong et al. (2022) reveal that the microbial biomass remains structurally stable for five growing seasons even under persistent stress imposed by grazing. In studying herbivore-plant-soil feedback in Mediterranean mountain grasslands, Castillo-Garcia et al. (2022) find that increasing grazing intensity increased soil available N, SOC, microbial biomass C, and beta-glucosidase activity.

2) Fertilizer N input

Increased quantity of soil N in grassland ecosystems often suppresses soil microbial biomass and modifies soil microbial functioning (Bai et al., 2020), due to the N-induced reduction in soil pH (Chen et al., 2019) altering biogeochemical cycling (Ning et al., 2021). The effect of N inputs depends on the rate and frequency of applications to the grassland. A higher N rate applied at a lower frequency decreases soil microbial biomass significantly, as the greater rate of N input causes C:N imbalance, decreasing the fungi-to-bacteria ratio. Also, the functions of soil microbial biomass may be influenced by soil N availability (Li W. T. et al., 2022). This influence of N input is modified by soil moisture level as water is needed to dissolve ${\text{NH}}_{4}^{+}$ - and ${\text{NO}}_{3}^{-}$ N (Li W. T. et al., 2022). Moisture availability influences several steps of N cycling in soil, including ammonia volatilization, nitrification, denitrification, and N leaching. Furthermore, the response of soil microbial biomass to N input is a result of complex interactions among climate, soil properties, and enzyme activity (Li Z. et al., 2019). In a low-N sandy grassland ecosystem, N input increases the amount of soil litter, which raised soil β-1,4-glucosidase activity and microbial biomass (Yayi et al., 2021).

3) Mowing

Mowing is a common grassland management practice, as the action enhances plant biodiversity (Ning et al., 2022) by reducing plant species richness (Yang et al., 2019), and increasing the availability of carbon for soil microorganisms (Wang Z. R. et al., 2019). However, the effect of mowing involves other factors. In a long-term study conducted in Western France, Gilmullina et al. (2020) report that mowing lowers SOC and decreases microbial biomass more than grazing. Removing plant biomass via mowing reduces C input to soil, limiting soil microbial biomass and soil enzyme production (Wang R. et al., 2020), which leads to decreased activity (Luo et al., 2019). However, inconsistent effects of mowing on plant-soil-microbe linkages are reported. For example, mowing increases the exposure of soil surface which increases soil temperature (Han et al., 2011) and decreases soil moisture (Shao et al., 2012), leading to decreased microbial biomass and enzyme activities (Mencel et al., 2022; Hopkins et al., 2023).

4) Grassland to cropland conversion

In the agro-pastoral transitional zone of northern China, the conversion of grassland to cropland leads to the loss of soil microbial biomass (Pan et al., 2023) and a shift in microbial community assembly, which is attributed to a decrease in SOM (Tang et al., 2022). The conversion from open grazing to enclosed grassland changes the input of nutrients affecting the size of soil microbial biomass (Pan et al., 2023). Grasslands are a complex plant-soil-microbe interactive ecosystem in which both enzyme activity and microbial biomass can be influenced by environmental conditions, including precipitation (Zhang and Xi, 2021), climate extremes such as severe drought (Holguin et al., 2022), and seasonal asynchrony between plant productivity and microbial biomass (Yin et al., 2022). Furthermore, conversion of grassland to cropland can lead to significant changes in soil mineralization (Li N. et al., 2022), nutrient cycling (Wang J. et al., 2020), SOC (Li J. et al., 2022), soil microbial biomass and community composition, and extracellular enzyme activity (Dong et al., 2021).

## 4 Apprehension and drawbacks

Many scholars foresee potential problems with the use of enzyme activities to assess soil fertility. The reliability of soil enzyme activities in predicting the degree of grassland ecosystem fertility is questioned. We summarize the following concerns and drawbacks:

1. Some scholars believe that soil enzyme activity changes rapidly and easily with a change in soil management practice. The status of enzyme activities in the soil is unstable, and hardly reflects the biological status of the soil fully, thereby, it is questionable about the accuracy of using enzyme activity to indicate soil fertility.
2. In some cases, the correlations between enzyme activity and soil fertility traits are quite weak or even not exist. For example, Pan et al. (2013) investigate the changes in soil physicochemical properties and enzyme activities and find that the activities of β-glucosidase and alkaline phosphatase are positively correlated with SOC, total N, and available N concentrations, but urease has a limited correlation with soil fertility. Increased soil fertility via N addition can promote the activity of C-cycling enzymes substantially while decreasing the activities of the other enzymes such as urease and alkaline phosphatase (Li Y. et al., 2021). Fertilizer-induced changes in microbial community characteristics (abundance, composition, diversity) can have far-reaching impacts on nutrient cycling and soil fertility.
3. The relation between soil fertility and enzyme activities may be confounded by soil management practices. For example, phosphate fertilizers depress phosphatase activity in soil; similarly, N fertilizers depress the enzymes involved in the N cycle (e.g., urease and amidase). Soil fertilized with N and P mineral fertilizers will show a much greater level of soil fertility than before being fertilized, but the relevant enzyme activities can be quite lower than before the fertilization. In addition can have a time-lag effect on enzyme activities, and increasing soil fertility through inorganic fertilization and residue input may have significantly positive impacts on some enzyme activities (Zhang et al., 2020). However, in some cases, the increased soil fertility can have no or negative impact on crop productivity by altering the abundance of dominant bacterial genera (Lan et al., 2023), or selectively enrich and inhibit the growth of certain bacterial taxa (Zhu et al., 2019). In these cases, using enzyme activity to indicate soil fertility may be an issue to interpret.
4. Some inconsistent or even opposite results on the relationships between enzyme activities and soil physiochemical properties have been reported. Claims are that the phenomenon may be related to environmental factors. Soil enzyme activities are shown to be much more sensitive to environmental factors, such as vegetation types and plant community composition, and drought stress, than soil physiochemical properties. ‘Sensitiveness’ is a good attribute for a soil fertility indicator, but enzyme activities may be too sensitive to temporal change in environmental conditions to be a reliable soil fertility indicator for the span of a cropping season, and too much influenced by environmental conditions for a global method to be developed.
5. In grassland ecosystem, the reaction of a specific enzyme is often influenced by the activities of other enzymes. The activity of specific enzymes in terms of the amount of substrate transformed or product produced is difficult to measure. Accordingly, the overall activity of enzymes may reflect the level of soil fertility only to a certain extent.

## 5 Suggestions to improve

To move forwards to the development of an alternative method to assess soil fertility, some scientists have made the following suggestions.

1. When the assay conditions change (e.g., temperature, moisture, pH, ionic strength), the enzyme activities will differ substantially. To make a meaningful assessment of soil fertility to compare different ecosystems, the protocol for measuring each enzyme assay needs to be standardized, including sampling procedures, sample pretreatment, assay procedures, and units of measurement of enzyme activities.
2. Some systematic studies across soil types, ecosystems, or long-term soil management sites are needed to identify the most appropriate enzyme assays to characterize specific soil parameters to reflect the level of soil fertility.
3. Standardized data sets need to be established, which can calibrate the enzyme assays and other soil properties, and interpretatesoil fertility indexes.
4. Other alternatives will be considered. For example, a soil fertility index can be established using a ‘minimum data set’ by measuring a bucket full of soil fertility-related parameters in a certain condition. Such an index can be used to reflect the level of soil fertility.

## 6 Conclusion and perspective

Enzyme activity can be considered an indicator of the status of soil biochemical properties. The enzyme assays are cost-efficient and operationally easier as compared to traditional soil fertility tests. Soil enzymes respond more rapidly to changes imposed on the ecosystem than the other soil fertility parameters, and thus assessing enzyme activities can provide timely information for decision-making on ecosystem management. However, enzyme activities in grassland ecosystem are affected by many factors, including climate change-induced stress and anthropogenic activities. The rapid response of enzymes to those factors may lead to inaccurate assessments of soil fertility which is an accumulative effect over a longer period. So far, methods of using enzyme activity to indicate soil fertility are still not ready. More solid, comprehensive evidence from multi-sites and multi-ecosystems is needed to validate the potential of the proposed indicator. Modelling needs to be done based on multi-site and multi-ecosystem data to validate the protocol of measuring the biological indicator of soil fertility.

## Author contributions

LW and GG conceived and designed the project. JW, DS, and YW conducted systematic reviews, and collected relevant data. PL and S-JL were involved in the draft manuscript. GG and LW wrote the manuscript, CH revised and edited the manuscript, and All authors contributed to the article and approved the submitted version.
